# Impact of preoperative factors and waiting time on post-appendectomy complications: a retrospective study

**DOI:** 10.1186/s13741-024-00365-z

**Published:** 2024-02-21

**Authors:** Shuo-Lun Lai, Chin-Hao Chang, Po-Chu Lee, Cheng-Maw Ho, Jin-Ming Wu, Hong-Shiee Lai, Been-Ren Lin

**Affiliations:** 1 Division of Colorectal Surgery, Department of Surgery, National Taiwan University Hospital, and National Taiwan University College of Medicine, No. 7, Chung-Shan South Road, Taipei, 100 Taiwan; 2 Department of Medical Research, National Taiwan University Hospital and National Taiwan University College of Medicine, Taipei, Taiwan; 3 Division of General Surgery, Department of Surgery, National Taiwan University Hospital, and National Taiwan University College of Medicine, Taipei, Taiwan; 4 Division of Pediatric Surgery, Department of Surgery, National Taiwan University Hospital and National Taiwan University College of Medicine, Taipei, Taiwan

**Keywords:** Appendicitis, Appendectomy, Delay, Complications, Timing

## Abstract

**Background:**

Several factors are associated with increased postoperative complications after appendectomies. However, few studies combined these potential factors for comprehensive prediction of surgical outcomes. Whether high-risk patients benefit from a shorter waiting time for surgery remains unclear. This study aimed to explore the impact of surgical waiting time and potential risk factors on postoperative complications.

**Methods:**

A total of 1343 patients diagnosed with acute appendicitis requiring an emergent appendectomy were included from 2013 to 2018. The preoperative risk factors associated with postoperative complications were selected and the probability of postoperative complications was calculated by multivariate logistic regression model. Patients were divided into four groups based on the risk (high & low) and time to surgery (> 12 & ≤12 hours). The odds ratios for complications were evaluated between groups.

**Results:**

The selected risk factors included age, neutrophil-lymphocyte ratio, systemic inflammatory response syndrome and abdominal pain duration. Compared with low-risk patients with time to surgery ≤12 hours, high-risk patients with time to surgery > 12 hours had significant increased overall postoperative complication rate (16.85% vs. 8.16%, *p* = 0.002) and a trend toward increased surgical site infection rate (10.99% vs. 6.46%, *p* = 0.058). When operated within 12 hours, there was no difference in outcomes between high- and low-risk patients. On the other hand, time to surgery > 12 hours did not increase complication rate in low-risk patients.

**Conclusions:**

The surgical outcome may be affected by preoperative factors and time to surgery. It is suggested that high-risk patients receive appendectomy within 12 hours to avoid increased postoperative complications.

**Supplementary Information:**

The online version contains supplementary material available at 10.1186/s13741-024-00365-z.

## Background

Acute appendicitis is one of the most common causes of an acute abdomen and usually requires emergent surgery. Although antibiotic treatment may be effective in select patients, appendectomy is still the only way to prevent recurrent appendicitis. Several factors (i.e., age, obesity, leukocytosis, symptom duration) have been proposed to be associated with an increased risk of postoperative complications (Andert et al. 2017; Kim et al. 2018b; Schlottmann et al. 2017). However, few studies combined these potential factors to predict the risk of surgical outcomes comprehensively.

Furthermore, there has been a dispute over the impact of surgical delay on outcomes for years. Some studies claimed that a prolonged waiting time for surgery increased the risk of complications (Ditillo et al. 2006; Giraudo et al. 2013) or surgical site infection (Teixeira et al. 2012), while others disagreed (Almstrom et al. 2017; Boomer et al. 2016; Boomer et al. 2014; van Dijk et al. 2018). If those patients at high risk for complication benefit from a shorter waiting time for appendectomies remains unclear. Since the coronavirus disease 2019 (COVID-19) became a pandemic and created challenges in managing surgical emergencies, optimizing the time to the operating room (OR) for select vulnerable patients has become an important issue. In this study, we aimed to develop a risk model for postoperative complications and identify patients who might be affected by surgical delay.

## Methods

### Patients and data collection

We retrospectively analyzed the surgical outcomes of patients diagnosed with acute appendicitis at a single tertiary medical center from 2013 to 2018. The Research Ethics Committee Office at National Taiwan University Hospital, Taipei, Taiwan approved this study (Institutional Review Board number: 202010066RINB). Patients who had visited our emergency room (ER) and underwent an emergent appendectomy were included in this study. Those who received conservative treatment first or had an elective appendectomy were excluded. Patients accidentally found appendicitis mixed with neoplastic lesions (i.e., adenocarcinoma, carcinoid, pseudomyxoma) or other conditions requiring combined surgery with other specialists (i.e., urologists, obstetricians, gynecologists) were excluded. Patients with incomplete emergency room records or an unclear history were also excluded.

Emergency room records included basic arrival information, clinical history, laboratory values, and imaging reports. Arrival information included the time of ER arrival, body temperature, pulse rate, respiratory rate, and blood pressure upon arrival at the ER. Perioperative data included surgery starting time, operative time, operative method (laparoscopic or open), operative findings, pathology reports, hospital stay, and postoperative complications. Abdominal pain duration, as an essential indicator in the study, was defined as the time from symptom onset to ER arrival. To minimize recall bias, we reviewed the historical record from the ER note, admission note, and nursing note. Cases with discordant descriptions were excluded. Time to surgery was defined as the time from ER arrival to surgery start.

### Treatment strategy, disease severity, and surgical outcomes

Empiric intravenous antibiotics were administered to all patients as soon as they were diagnosed with acute appendicitis by clinical or imaging findings. Unless contraindicated, the preferred image study is computed tomography (CT) because of its reduced waiting time in recent years. The CT images helped surgeons evaluate disease severity and accessibility of surgery. The decision for surgery was based on the surgeon’s opinion and consensus with the patients. Patients who agreed to appendectomy were placed on the emergent operation waiting list. At our institution, the emergent operations are continuously executed regardless of day/night shift, but the operation room usage is reduced at night. The order of emergent surgery is affected by the degree of urgency.

Disease severity was classified as perforation and non-perforation based on intraoperative and pathological findings. An abscessed or ruptured appendix were defined as perforation. The gangrenous change was defined as non-perforation because the appendiceal wall remained intact. There was evidence that treating gangrenous appendicitis as a non-perforated disease did not affect surgical outcome (Nordin et al. 2019). The correlation of disease severity and postoperative complications were evaluated using the Chi-square test.

Postoperative complications included surgical site infection, intra-abdominal abscess formation, ileus, and internal bleeding. Surgical site infection referred to an incisional wound infection, and intra-abdominal abscess referred to a deep infection from the appendectomy area. Patients with surgical site infections required incision and drainage, wet dressings, or a special sterile dressing on their wounds with oral or intra-venous antibiotic administration. An intra-abdominal abscess was confirmed on computed tomography when patients reappeared to the ER complaining of a fever or abdominal pain after discharge. Ileus was diagnosed if the patient required short-term nasogastric tube decompression or prokinetic drugs during recovery. Postoperative internal bleeding requires emergent surgical intervention or transarterial embolization. Inpatient and outpatient records of all patients were followed, and patients with adverse events after surgery were graded with Clavien-Dindo Classification.

### Preoperative risk and time to surgery

The clinical characteristics of patients with complications and without complications were compared using a Chi-square or Fisher’s exact test for categorical variables and an independent Student’s t-test for continuous variables. The preoperative factors were analyzed by univariate logistic regression. Afterward, the significant factors (*p* < 0.05) were included in the multivariate model. The probability of postoperative complications for all patients was calculated by the multivariate model. Patients with probability higher than the median were classified as high risk, and patients with probability lower than the median were classified as low risk.

An interim guideline for timing of emergent surgery was proposed by the Timing of Acute Care Surgery Classification (TACS) study group. Although the guideline recommended appendectomy within 12 hours, expert opinion and literature reviews varied on the timing of appendectomy (Kluger et al. 2013). Patients were further divided into four groups: 1) low-risk patients with time to surgery ≤12 hours, 2) high-risk patients with time to surgery ≤12 hours, 3) low-risk patients with time to surgery > 12 hours, and 4) high-risk patients with time to surgery > 12 hours. The odds ratios for postoperative complications between four groups were estimated using the logistic regression.

In addition, for continuous variables that were significantly related with postoperative complications, a cut-off point was obtained by Youden index using receiver operating characteristic (ROC) curve. The results were shown in supplementary data. All statistical analysis was performed with SAS version 9.4 (SAS Institute Inc., Cary, NC). Statistical significance was defined as *p* < 0.05.

## Results

**Fig. 1:**
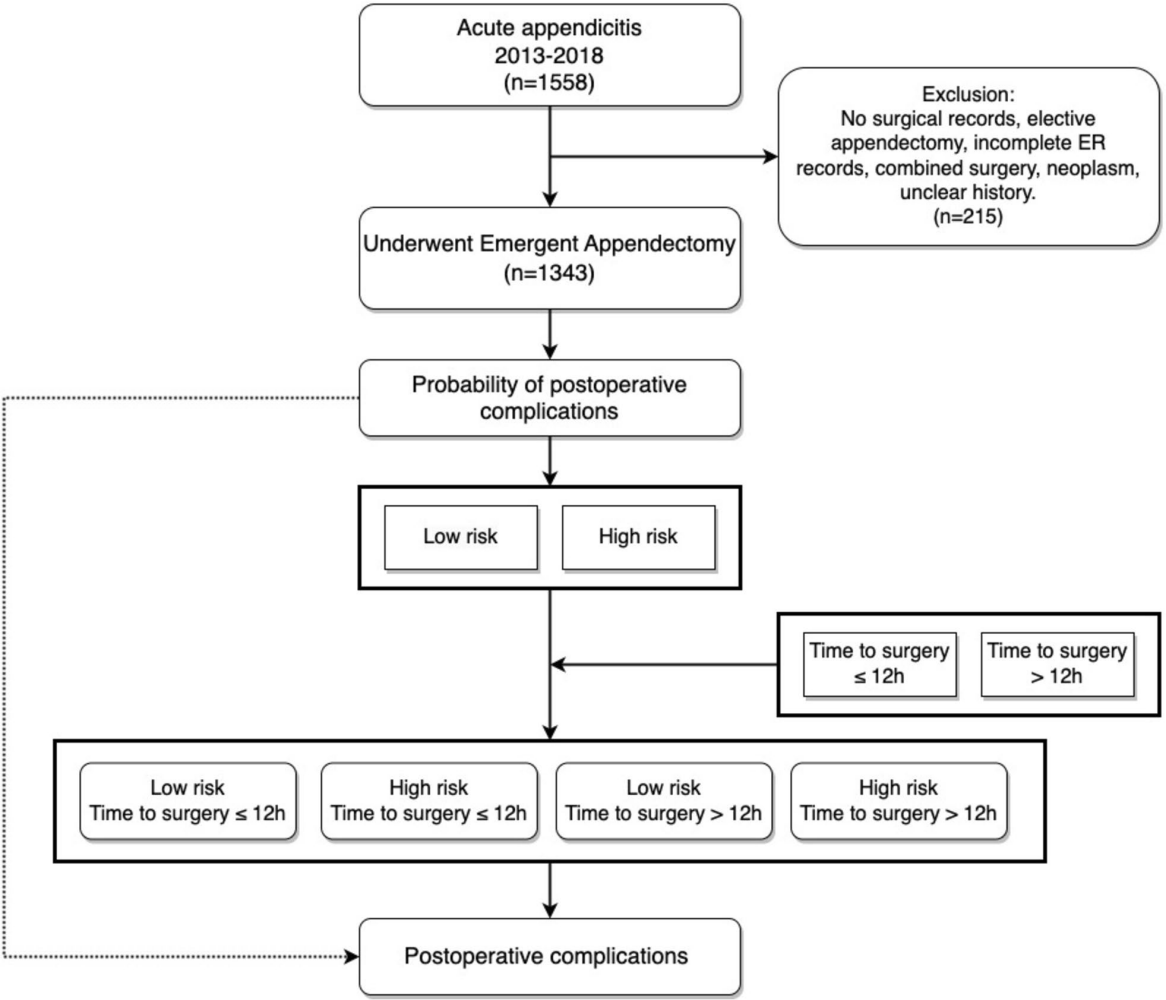
Study flow chart

From 2013 to 2018, a total of 1558 patients diagnosed with acute appendicitis were cared for at our institution, of whom 1343 were included in this study. The study flowchart is shown in Fig. 1.

### Statistics of postoperative complications after emergent appendectomy

Overall, complications occurred in 130 patients after surgery (Table 1). Among them, 88 (6.55%) patients had surgical site infections, 20 (1.49%) had an intraabdominal abscess, 32 (2.38%) had ileus, and 1 (0.07%) had internal bleeding. In the Clavien-Dindo classification, Grade I, Grade II, and Grade III were 16 (12.3%), 108 (83.08%), and 6 (4.62%), respectively. No complication was graded as Grade IV.

**Table 1:** Patients with postoperative complications (n, %)

Overall complications	130 (9.68%)
Surgical site infection	88 (6.55%)
Intra-abdominal abscess	20 (1.49%)
Ileus	32 (2.38%)
Internal bleeding	1 (0.07%)
Clavien-Dindo Classification	
Grade I	16 (12.3%)
Grade II	108 (83.08%)
Grade III	6 (4.62%)
Grade IV	0 (0%)

The baseline demographic and clinical characteristics of patients with or without postoperative complications are shown in Table 2. The complication rate was 9.68%, which was similar to other studies (Andert et al. 2017; Kim et al. 2018b). The average diagnostic time was 3.86 hours, indicating that patients were administered empiric antibiotics within 4 hours. There were significant differences in age, abdominal pain duration, operative time, systemic inflammatory response syndrome (SIRS), hospital stay, and disease severity between groups. Perforation rate was higher in complication group (52.31% vs. 27.45%, *p* < 0.001) in terms of disease severity. There was a trend toward a higher neutrophil-lymphocyte ratio (NLR) in the complication group (11.94 vs. 10.32), but it did not reach statistical significance (*p* = 0.055).

**Table 2:** Baseline demographics and clinical characteristics

	All Patients (*n* = 1343)	Without complications (*n* = 1213)	With complications (*n* = 130)	*p*-value
Age (Mean, SD)	38.25 (19.33)	37.7 (19.28)	43.45 (19.05)	0.001^a^
Sex (n, %)				0.212^b^
Female	638 (47.51%)	583 (48.06%)	55 (42.31%)	
Male	705 (52.49%)	630 (51.94%)	75 (57.69%)	
Body Mass Index (Mean, SD)	22.86 (4.59)	22.82 (4.66)	23.19 (3.89)	0.315^a^
Body temperature at triage (n, %)				0.235^b^
36–38 °C	1095 (81.53%)	994 (81.95%)	101 (77.69%)	
< 36 or > 38 °C	248 (18.47%)	219 (18.05%)	29 (22.31%)	
Heart rate at triage (n, %)				0.121^b^
≤ 90 bpm	655 (48.77%)	600 (49.46%)	55 (42.31%)	
> 90 bpm	688 (51.23%)	613 (50.54%)	75 (57.69%)	
Respiratory rate at triage (n, %)				0.299^b^
≤ 20 /min	1216 (90.54%)	1095 (90.27%)	121 (93.08%)	
> 20 /min	127 (9.46%)	118 (9.73%)	9 (6.92%)	
Systolic blood pressure at triage (Mean, SD)	124.2 (20.32)	124.12 (20.25)	124.98 (21.08)	0.645^a^
Diastolic blood pressure at triage (Mean, SD)	75.4 (22.02)	75.51 (22.68)	74.4 (14.47)	0.437^a^
Pain score (VAS) (Mean, SD)	5.98 (1.81)	5.98 (1.76)	6 (2.21)	0.928^a^
Abdominal pain duration (n, %)				0.001^b^
< 24 h	708 (52.72%)	659 (54.33%)	49 (37.69%)	
24-48 h	273 (20.33%)	241 (19.87%)	32 (24.62%)	
> 48 h	362 (26.95%)	313 (25.8%)	49 (37.69%)	
Diagnostic tool				1.000^c^
Physical examination	2 (0.15%)	2 (0.17%)	0 (0%)	
CT	1315 (98.95%)	1187 (98.92%)	128 (99.22%)	
Ultrasound	10 (0.75%)	9 (0.75%)	1 (0.78%)	
MRI	2 (0.15%)	2 (0.17%)	0 (0%)	
Diagnostic time (ER- > Exam) (h) (Mean, SD)	3.86 (2.79)	3.88 (2.8)	3.68 (2.68)	0.443^a^
Time to surgery (ER- > OR) (h) (n, %)				0.536^b^
≤ 12 h	593 (51.84%)	534 (52.15%)	59 (49.17%)	
> 12 h	551 (48.16%)	490 (47.85%)	61 (50.83%)	
Operative time (min) (Mean, SD)	70.99 (32.46)	70.18 (32.58)	77.85 (30.68)	0.014^a^
Operative method (n, %)				0.074^c^
Laparoscopic	1324 (98.73%)	1198 (98.93%)	126 (96.92%)	
Open	17 (1.27%)	13 (1.07%)	4 (3.08%)	
White blood cell count (n, %)				0.677^b^
4000-12,000	456 (33.95%)	414 (34.13%)	42 (32.31%)	
< 4000 or > 12,000	887 (66.05%)	799 (65.87%)	88 (67.69%)	
Neutrophil-Lymphocyte Ratio (Mean, SD)	10.47 (7.85)	10.32 (7.69)	11.94 (9.12)	0.055^a^
Platelet-Lymphocyte Ratio (Mean, SD)	27.87 (21.39)	27.64 (21.45)	30.04 (20.73)	0.229^a^
Systemic inflammatory response syndrome				0.037^b^
No	716 (53.31%)	658 (54.25%)	58 (44.62%)	
Yes	627 (46.69%)	555 (45.75%)	72 (55.38%)	
Hospital stay in days (Mean, SD)	3.7 (3.45)	3.35 (2.09)	7 (8.4)	< 0.001^a^
Disease severity				< 0.001^b^
Non-perforation	942 (70.14%)	880 (72.55%)	62 (47.69%)	
Perforation	401 (29.86%)	333 (27.45%)	68 (52.31%)	

^a^Estimated by independent t test

^b^Estimated by chi-square test

^c^Estimated by Fisher’s exact test (Non-parameter statistic)

### Risk for postoperative complications

Of the clinical characteristics listed in Table 2, potential risk factors for postoperative complications were selected. On univariate analysis, age, NLR, SIRS, and abdominal pain duration 24-48 hr. and > 48 hr. had a statistically significant relationship with postoperative complications. On multivariate analysis, age and abdominal pain > 48 h reached statistical significance (Table 3).

**Table 3:** Association of preoperative factors and postoperative complications

	Univariate model		Multivariate model	
	OR (95% CL)	*p*-value	OR (95% CL)	*p*-value
Age	1.0152 (1.0059,1.0247)	0.001	1.0153 (1.0059,1.0249)	0.001
Sex	1.2619 (0.8754,1.8191)	0.213		
Body Mass Index	1.0162 (0.9803,1.0534)	0.382		
Body temperature at triage				
36–38 °C	ref			
< 36 or > 38 °C	1.3034 (0.8411,2.0199)	0.236		
Heart rate at triage				
≤ 90 bpm	ref			
> 90 bpm	1.3347 (0.9259,1.924)	0.122		
Respiratory rate at triage				
≤ 20 /min	ref			
> 20 /min	0.6902 (0.3416,1.3945)	0.302		
Systolic blood pressure at triage	1.0021 (0.9933,1.011)	0.645		
Diastolic blood pressure at triage	0.9964 (0.9843,1.0088)	0.570		
Pain score (VAS)	1.0056 (0.9096,1.1117)	0.914		
Diagnostic time (ER- > Exam) (h)	0.9704 (0.8985,1.048)	0.444		
Time to surgery (ER- > OR) (h)				
≤ 12 h	ref			
> 12 h	1.1267 (0.7718,1.6448)	0.536		
White blood cell count				
4000-12,000	ref			
< 4000 or > 12,000	1.0856 (0.7378,1.5975)	0.677		
Neutrophil-Lymphocyte Ratio	1.0232 (1.0025,1.0444)	0.028	1.0192 (0.9976,1.0413)	0.082
Platelet-Lymphocyte Ratio	1.0046 (0.9971,1.0121)	0.231		
Systemic inflammatory response syndrome	1.4718 (1.0229,2.1175)	0.037	1.4499 (0.9791,2.1472)	0.064
Abdominal pain duration				
< 24 h	ref		ref	
24-48 h	1.7858 (1.1169,2.8551)	0.015	1.6047 (0.9928,2.5937)	0.054
> 48 h	2.1054 (1.3859,3.1986)	0.001	1.9907 (1.2955,3.0588)	0.002

Age, NLR, SIRS, and abdominal pain duration 24-48 hr. and > 48 hr. were selected as parameters of a preoperative probability calculation. Based on the multivariate logistic regression model, the probability was calculated as follows:

$$Score=\left(-3.5807\right)+ Age\times 0.0152+ NLR\times 0.0190+ SIRS\times 0.3715+ Duration\left(24-48 hr\right)\times 0.4729+ Duration\left(>48 hr\right)\times 0.6685$$

$$Probability\ of\ postoperative\ complications=\frac{\exp (Score)}{1+\exp (Score)}$$

The median probability was 0.084748 for all patients. Patients with a probability higher than 0.084748 were classified as high risk. Patients with a probability lower than 0.084748 were classified as low risk. Overall, high-risk patients had significant higher complication rate (14.23% vs. 6.55%, *p* < 0.001) and surgical site infection rate (9.07% vs. 5.13%, *p* = 0.01) than low-risk patients.

### The interactive effect of preoperative risk and time to surgery on surgical outcomes

After omitting patients with missing perioperative time data, 583 patients were operated within 12 hours and 544 patients were operated after more than 12 hours. The four-group analysis is shown in Table 4. Compared with low-risk patients with time to surgery ≤12 hours, high-risk patients with time to surgery > 12 hours had significant increased overall postoperative complication rate (16.85% vs. 8.16%, *p* = 0.002) and a trend toward increased surgical site infection rate (10.99% vs. 6.46%, *p* = 0.058). When operated within 12 hours, there was no difference in complication rate (11.76% vs. 8.16%, *p* = 0.148) or surgical site infection rate (7.27% vs. 6.46%, *p* = 0.701) between high- and low-risk patients. On the other hand, time to surgery > 12 hours did not increase complication rate (4.8% vs. 8.16%, *p* = 0.110) or surgical site infection rate (3.69% vs. 6.46%, *p* = 0.141) in low-risk patients.

**Table 4:** Effect of preoperative risk and time to surgery on postoperative complications

Group	Time to surgery ≤12 h Low risk (294)	Time to surgery ≤12 h High risk (289)	Time to surgery > 12 h Low risk (271)	Time to surgery > 12 h High risk (273)
Overall complications	24 (8.16%)	34 (11.76%)	13 (4.80%)	46 (16.85%)
Odds ratio (95%CI)	ref	1.5 (0.8656,2.5995)	0.567 (0.2826,1.1374)	2.2798 (1.3498,3.8505)
*p*-value		0.148	0.110	0.002
Surgical site infection	19 (6.46%)	21 (7.27%)	10 (3.69%)	30 (10.99%)
Odds ratio (95%CI)	ref	1.1341 (0.5962,2.1573)	0.5546 (0.2532,1.2149)	1.7869 (0.9807,3.2559)
*p*-value		0.701	0.141	0.058

## Discussion

There was wide disagreement about how time to surgery influences surgical outcomes (Abdul Jawad et al. 2021; Almstrom et al. 2017; Boomer et al. 2016; Boomer et al. 2014; Ditillo et al. 2006; Drake et al. 2014; Giraudo et al. 2013; Kearney et al. 2008; Meltzer et al. 2019; Teixeira et al. 2012; van Dijk et al. 2018), but few studies have attempted to identify specific patients potentially affected by the surgical delay. Several scoring or grading systems for predicting surgical outcomes were proposed, but most required intraoperative findings (Emile et al. 2021; Finnesgard et al. 2018; Noorit et al. 2018; Vasileiou et al. 2019), which means those systems could not help reverse these complications. Our study used a multivariate logistic regression model consisting of preoperative factors to classify patients into high and low risk. Although patients in our study received intravenous antibiotics within 4 hours on average after admission to ER, high-risk patients with a surgical delay of > 12 h had significantly higher complications and surgical site infection rates than low-risk patients. In contrast, outcomes were similar when surgery was performed within 12 hours. In recent years, treatment strategies for acute appendicitis have changed. Conservative treatment for appendicitis was successful in uncomplicated patients (Collaborative et al. 2020; Podda et al. 2019; Sallinen et al. 2016). When dealing with perforated appendicitis, a meta-analysis study revealed that patients might benefit from antibiotics or drainage prior to surgery despite heterogeneity in findings (Simillis et al. 2010). Nevertheless, some of the patients might eventually still need an appendectomy due to recurrence. At our institution, CT became a standard tool in ER because CT helps not only diagnose the disease but also differentiate disease severity (Bixby et al. 2006; Horrow et al. 2003; Kim et al. 2018a). In the era of the COVID-19 pandemic, it is more important than ever to accurately prioritize emergent surgeries in order to preserve our medical resources, including hospital staff and operating room schedules. A recent study reported that prolonged time to consultation due to COVID-19 quarantine might increase rates of severe peritonitis and intra-abdominal abscess formation (Dreifuss et al. 2020). Risk for postoperative complications can be calculated immediately once the patient is diagnosed with acute appendicitis. Our study suggests that low-risk patients are safe for surgical delay > 12 h under the administration of intravenous antibiotics. On the other hand, high-risk patients are advised to receive emergent surgery within 12 hours to avoid increased complications and surgical site infection rate.

The parameters of the preoperative probability calculation included age, NLR, presence of SIRS, and abdominal pain duration. Research had shown that aging patients had slower wound healing and increased risk of surgical site infection (Engeland et al. 2006; Lizán-García et al. 1997). Jawad et al. also stated that older age and a higher Charlson Comorbidity Index were related to appendicitis progression and worse outcomes (Abdul Jawad et al. 2021). Several studies reported that NLR was related to disease severity (Ishizuka et al. 2012; Shimizu et al. 2016) and thus might influence postoperative complications. We hypothesized that the presence of SIRS/sepsis was indicative of disease progression from a localized to systemic infection and therefore associated with postoperative complications. Boomer et al. (Boomer et al. 2016) echoed our hypothesis, showing that the incidence of sepsis was significantly higher in the surgical site infection group. Studies had shown that abdominal pain duration was an essential indicator for disease severity (Dreifuss et al. 2020; Oliak et al. 2000; Lai et al. 2018) and surgical outcomes (Kim et al. 2018b), which was consistent with our results since abdominal pain duration is a significant factor in the probability calculation.

There are several limitations to this study. First, it was a single-institute retrospective study, and the variation between different institutes should be considered. Further multi-institution prospective studies or population-based analysis may help validate our research. Second, the study did not include CT results due to variability in reports style between different radiologists. Furthermore, the decision for surgery was usually made before the official report was uploaded. In the future study, we will cooperate with radiologists to identify specific CT features associated with postoperative complications and add them to the prediction. Third, although most preoperative characteristics were factual data, recall bias was inevitable when recording abdominal pain duration. To limit this impact, symptom duration was triple-checked from the ER record, admission note, and nursing note. Cases with discordant descriptions were excluded. Fourth, the operator’s surgical experience is a non-negligible factor for postoperative complications but was not measurable in the study (Scarborough et al. 2012). Resident participation in the surgery and patient care should be considered in a future study.

## Conclusions

The preoperative risk is a helpful indicator for surgeons to select vulnerable patients and arrange surgery in an appropriate time frame. The prolonged waiting time for surgery does not lead to worse surgical outcomes for low-risk patients. In contrast, high-risk patients are recommended to undergo appendectomy within 12 hours to avoid increased complications.

### Supplementary Information

**Supplementary material 1.**

## Data Availability

The datasets used and/or analyzed during the current study are available from the corresponding author on reasonable request.

## Abbreviations

COVID-19: Coronavirus disease 2019
OR: Operating room
ER: Emergency room
CT: Computed tomography
TACS: Timing of acute care surgery classification
SIRS: Systemic inflammatory response syndrome
NLR: Neutrophil-lymphocyte ratio
