# Supplementary material for: Impact of preoperative factors and waiting time on post-appendectomy complications: a retrospective study
Source: Perioper Med (Lond). 2024 Feb 21;13:8. doi: 10.1186/s13741-024-00365-z (PMC10880317; doi:10.1186/s13741-024-00365-z)

Supplementary figure 1. Receiver operating characteristic curve and cut-off value for significant continuous variables. (A) The area under curve for age is 0.59 and the cut-off value is 31 years old. (B). The area under curve for Neutrophil-Lymphocyte Ratio (NLR) is 0.54 and the cut-off value is 5.9. On univariate analysis, categorical NLR (≥ 5.9 vs. < 5.9) didn’t reach statistically significant difference (p=0.122) for postoperative complications.


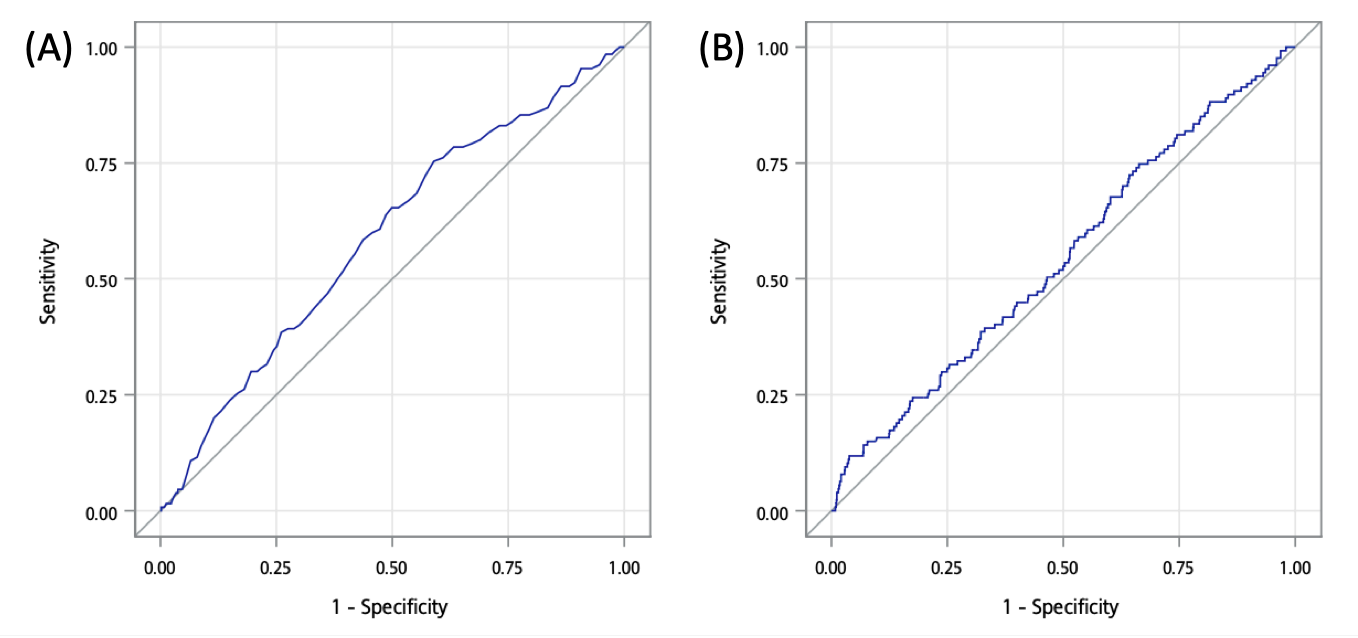

Supplement: Supplementary file 1 — Supplementary material 1. [file 13741_2024_365_MOESM1_ESM.docx]
